# A promising cellulose-based polyzwitterion with pH-sensitive charges

**DOI:** 10.3762/bjoc.10.159

**Published:** 2014-07-08

**Authors:** Thomas Elschner, Thomas Heinze

**Affiliations:** 1 Center of Excellence for Polysaccharide Research, Institute for Organic Chemistry and Macromolecular Chemistry, Friedrich Schiller University of Jena, Humboldtstraße 10, D-07743 Jena, Germany

**Keywords:** carbonate, cellulose, complexation, multivalent glycosystems, NMR, polyzwitterion

## Abstract

A novel polyzwitterion possessing weak ionic groups could be efficiently synthesized from cellulose phenyl carbonate. Polyanion, polycation, and polyzwitterion are accessible by orthogonal removal of protecting groups. The molecular structure was proofed by FTIR- and NMR spectroscopy. Characteristic properties of the cellulose derivatives, e.g., acid dissociation constants, isoelectric point and complexation, were investigated by potentiometric titration (pH), nephelometry, rheology and dynamic light-scattering. The formation of pH-responsive interpolyelectrolyte complexes applying polydiallyldimethylammonium chloride was preliminary studied.

## Introduction

Ionic polymers are important naturally occurring macromolecules and various synthetic polyelectrolytes play an important role in commercial applications [1]. Naturally occurring biopolymers are for example proteins, nucleotides, and polysaccharides like alginates. Synthetic ionic polymers are widely used in the field of water treatment [2], protein separation, desalination, binding of metal ions, in the oil industry [3] and nanotechnology [4]. In general, these polymers may be divided into water-soluble polyelectrolytes, containing anionic or cationic groups, and polyzwitterions that include both charges. While the addition of low molecular weight electrolyte cause the polyelectrolyte effect, i.e., the shrinking and precipitation of the polyelectrolyte, polyzwitterions show chain expansion and increased solubility (antipolyelectrolyte effect). Moreover, the classification may result from weak and strong ionic groups, and the location of the charges. Polyampholytes possess the charged groups on different monomer units, while polybetaines refer to polymers with opposite charges on one side chain at the same monomer unit [1].

The first statistical, synthetic polyampholytes were obtained in the 1950s by radical polymerization of acrylic- or methacrylic acid and their derivatives [5–7]. 20 years later the first block polyampholytes were synthesized via anionic polymerization of 2-vinylpyridine with trimethylsilyl methacrylate [8–9]. Polybetaines, for example sulfobetaines, could be prepared from the reaction of a tertiary amine (monomer or polymer) and a sultone [10]. Due to their biocompatibility and hemocompatibility polybetaines are attributed to be materials with biomimetic properties [11]. However, polybetaines are mostly not soluble in pure water. Their solubility is often limited to concentrated saline solutions or organic solvents with high hydrogen bond-donating ability (e.g., trifluoroethanol) [12]. Therefore, the processing to yield films or nanoparticles requires ecologically harmful solvents. Thus, a broad variety of synthetic polyampholytes and polybetaines were synthesized, however, they are not soluble in pure water.

The chemical modification of polysaccharides allows the access to novel polyzwitterions where the opposite charges may be located in the same repeating unit, but not in the same side chain. Moreover, regioselective functionalization is possible [13]. In contrast to synthetic polybetaines an increased solubility could be expected for polysaccharide derivatives and, furthermore, biopolymers possess an inherent biocompatibility and biodegradability. While polyzwitterions based on N-functionalized chitosan [14–15] or 6-desoxy-6-aminocelluloses [16] are composed like synthetic polybetaines, a few cellulose-based zwitterions are described where the isoelectric point and the properties in solution could be tuned by varying the substitution pattern [17–20]. However, the polymers contain mostly a permanent cationic- or anionic charge, which may lead to limitations in solubility. Thus, there is an increasing interest in novel biobased, zwitterionic polysaccharide derivatives in research and industry.

In the present work the structure design of novel polyzwitterions based on cellulose carbamate with carboxylic- and primary amino groups is discussed. Starting from cellulose carbonate, the fully protected zwitterion is efficiently synthesized that could be converted into the polyanion, polycation, and polyzwitterion via the orthogonal removal of the protecting groups. The molecular structure of the products is investigated by means of FTIR- and NMR spectroscopy in detail. Moreover, important properties including the acid dissociation constants, the isoelectric point and the aggregation behavior are determined by potentiometric titration (pH), nephelometry, rheology and dynamic light-scattering. Moreover, preliminary studies about stimuli-responsive interpolyelectrolyte complexes are presented.

## Results and Discussion

### Synthesis

**Figure 1:**
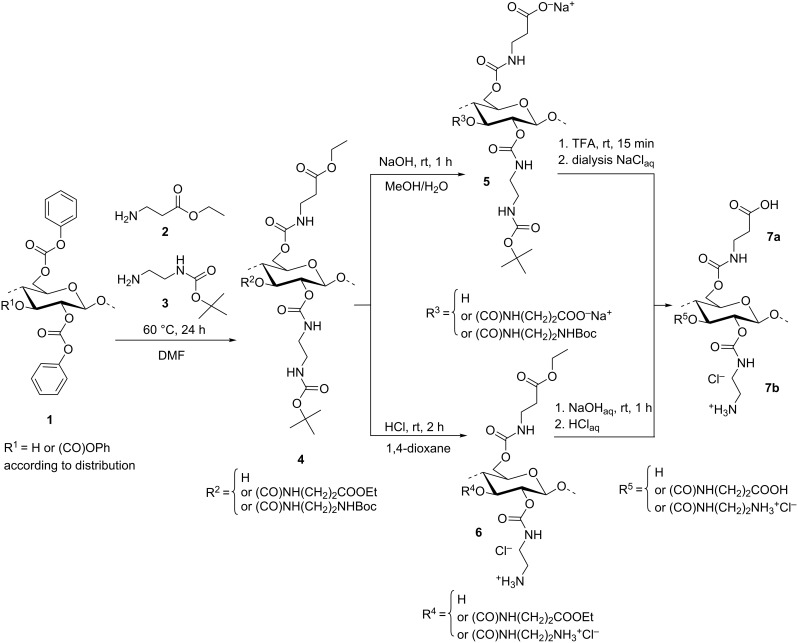
Reaction scheme of the synthesis of anionic, cationic, and ampholytic cellulose carbamates (substituents randomly distributed).

The synthesis of a novel polyzwitterion based on cellulose was performed by activation of the polymer backbone by formation of a carbonate moiety and subsequent introduction of amino- and carboxylic groups using the protected diamine and amino acid. As schematically shown in Figure 1, cellulose phenyl carbonate (**1**, degree of substitution, DS 1.92), obtained by homogeneous conversion of cellulose with phenyl chloroformate in *N*,*N*-dimethylacetamide (DMAc)/LiCl and pyridine [21], was allowed to react with an equimolar mixture of β-alanine ethyl ester (**2**) and *N*-*tert*-butoxycarbonyl-1,2-ethanediamine (**3**). The β-alanine ethyl ester was obtained from the respective commercially available hydrochloride under triethylamine deficiency conditions in order to avoid cross-linking of the polymer chains via carbonate moieties resulting from the strong basicity of the trialkylamine [22]. The aminolysis yields the (3-ethoxy-3-oxopropyl)(*N*-Boc-2-aminoethyl)cellulose carbamate **4** with a DS_β-alanine ester_ of 0.88 and a DS_Boc-EDA_ of 0.95 that indicates a similar reactivity of the amines applied and the conversion of 95% of the carbonate moieties into the carbamate. Moreover, a random distribution of the substituents within the repeating unit and the polymer backbone can be assumed.

From cellulose carbamate **4**, the polyanion **5**, the polycation **6**, and the polyzwitterion **7a**,**b** may be obtained because of the orthogonal protecting groups. The homogeneous alkaline cleavage of the ethyl ester into (2-carboxyethyl)(*N*-Boc-2-aminoethyl)cellulose carbamate **5** was carried in methanolic/aqueous sodium hydroxide solution to mediate the solubility of educt and product. The acidic cleavage of the Boc group applying gaseous hydrogen chloride leads to the polycation **6**.

Finally, the polyzwitterion (2-carboxyethyl)(2-aminoethyl)cellulose carbamate **7a** could be obtained by acidic treatment of polyanion **5**. However, the solubility in aprotic organic solvents, like dioxane and CH_2_Cl_2_, is decreased and the deprotection has to be carried out in trifluoroacetic acid (TFA), followed by a subsequent anion exchange by dialysis of the polymer solution against aqueous sodium chloride and water. **7b** was prepared from the polycation **6** by alkaline treatment, but the acidification of the reaction mixture is required for a convenient isolation of the product **7b**.

### Structure characterization

**Figure 2:**
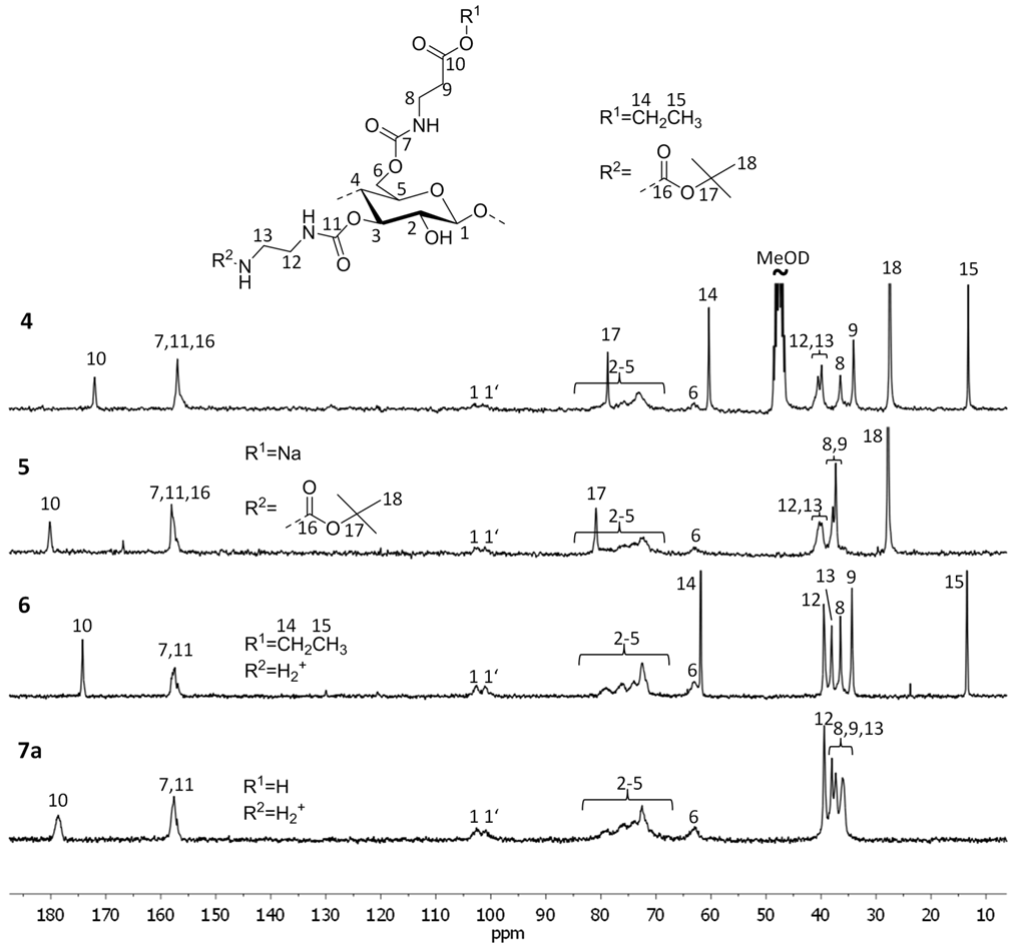
^13^C NMR spectra of (3-ethoxy-3-oxopropyl)(*N*-Boc-2-aminoethyl)- (**4**), (2-carboxyethyl)(*N*-Boc-2-aminoethyl)- (**5**), (3-ethoxy-3-oxopropyl)(2-aminoethyl)- (**6**) and (2-carboxyethyl)(2-aminoethyl)cellulose carbamate (**7a**) recorded in MeOD (**4**) or D_2_O (**5**–**7a**).

The molecular structure of the products obtained could be revealed by means of FTIR- and NMR spectroscopy. The C=O stretching vibration of the ester- and the carbamate moiety overlap in the IR spectrum to a broad signal at 1716 cm^−1^. The carboxylate in polyanion **5** can be detected separately from the carbamate linkage (1705 cm^−1^) at 1581 cm^−1^. While the C=O peaks in the IR spectrum of the polyzwitterion, arising from the carbamate and the carboxylic group, cannot be resolved, NMR spectroscopy may be applied for detailed structure characterization. Figure 2 shows the ^13^C NMR spectra of the (3-ethoxy-3-oxopropyl)(*N*-Boc-2-aminoethyl)- (**4**), the anionic- (**5**), the cationic- (**6**), and the zwitterionic (**7a**) cellulose carbamate. The C=O resonances at 172 and 157 ppm indicate the ester and the carbamate moieties, respectively. Signals arising from the cellulose backbone are visible at 103–101 (C-1), 83–74 (C-2-5) and 63 ppm (C-6). Moreover, the resonances of aliphatic carbon nuclei of the substituents occur at 78.8 (CMe_3_), 60.4 (OCH_2_), 40.5–34.1 (CH_2_), 27.5 (C(CH_3_)_3_), and 13.2 ppm (CH_3_). The cleavage of the ethyl ester to the corresponding carboxylate **5** is indicated by a shift of the C=O resonance to lower field. Moreover, the signals belonging to the ethyl group disappear and the peak of the methylene moiety next to the carboxylate is shifted to low field of about 3 ppm. The removal of the Boc group leads to the cation **6** clearly visible by the absence of the resonances at 78 and 28 ppm. After the cleavage of both, the ethyl ester and the Boc moiety, the polyzwitterion **7a** is obtained indicated by the missing resonances. Furthermore, the desired functionalities are still linked to the polymer backbone, proofed by C=O resonances, signals from position 1 to 6 of the repeating unit, and the peaks of four CH_2_ groups.

### Properties

#### Acid dissociation constants and isoelectric point

**Figure 3:**
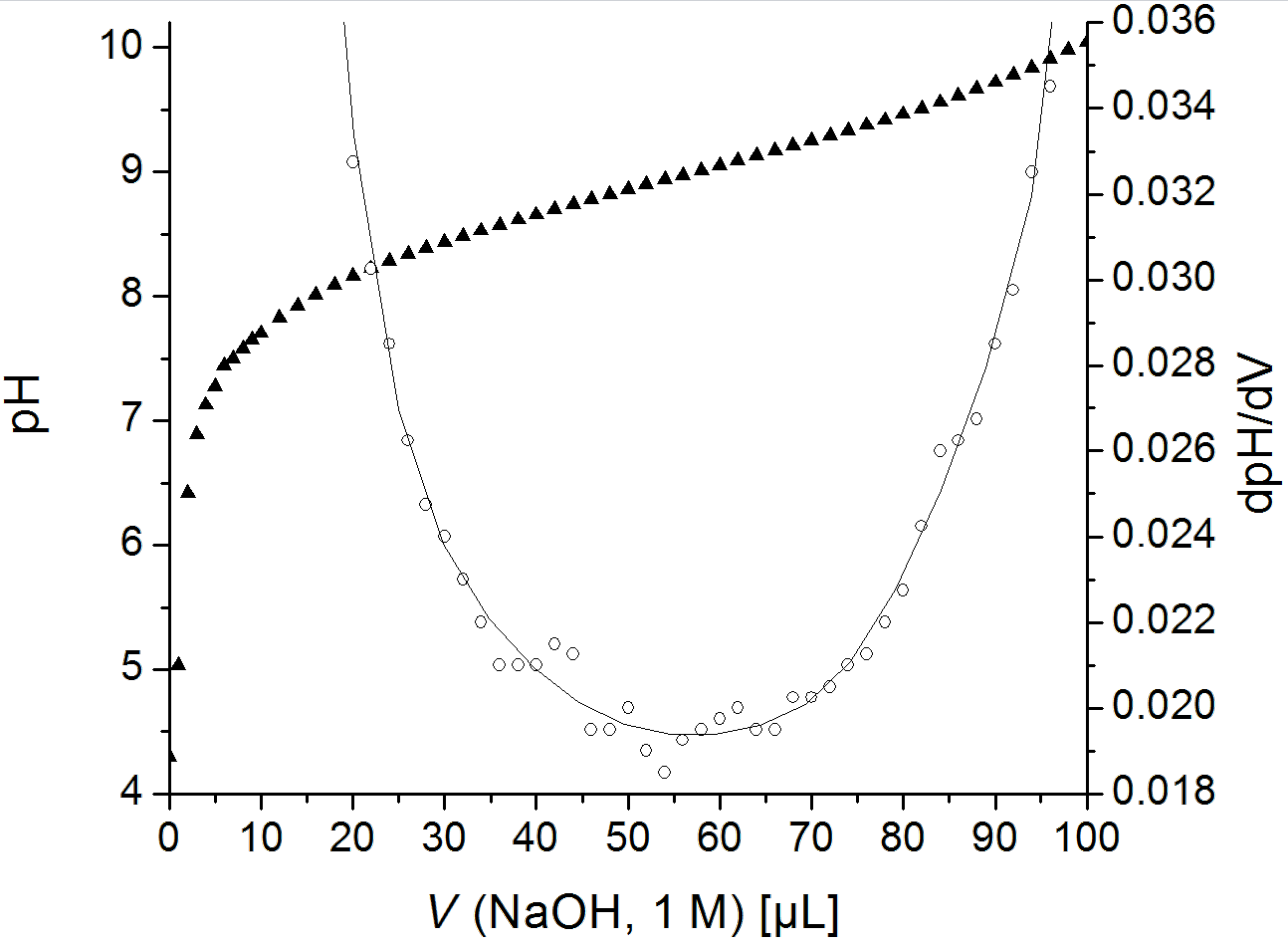
Acid–base titration of (3-ethoxy-3-oxopropyl)(2-aminoethyl)cellulose carbamate (**6**, 0.4%) in 0.2 M aqueous sodium chloride solution: pH (*V* NaOH) ▲ and its first derivative dpH/dV ○.

**Figure 4:**
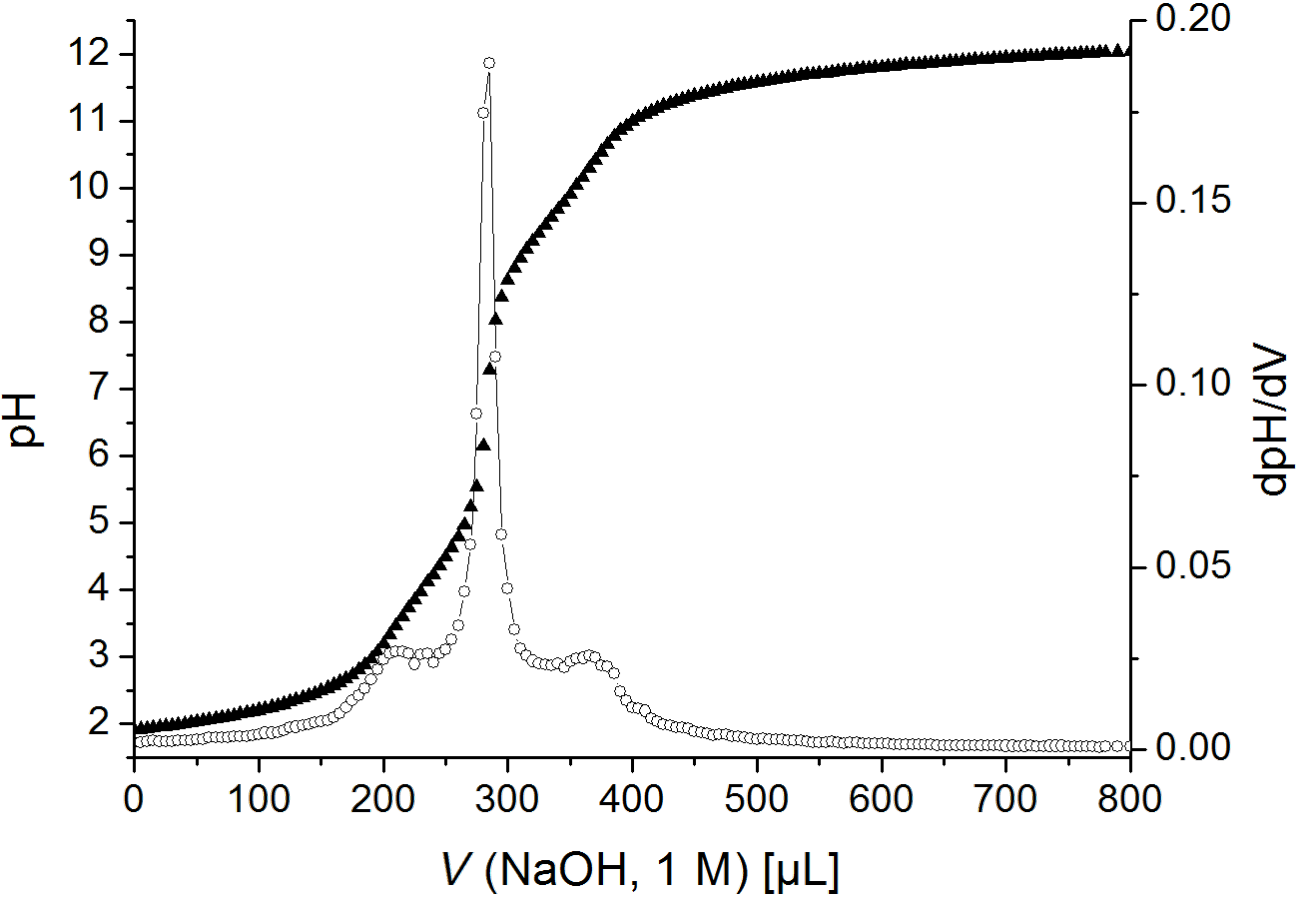
Acid–base titration of (2-carboxyethyl)(2-aminoethyl)cellulose carbamate (**7a**, 0.4%) in 0.2 M aqueous sodium chloride solution: pH (*V* NaOH) ▲ and its first derivative dpH/dV ○.

The acid dissociation constants could be determined by potentiometric titration (pH). Moreover, the question arises how the p*K*_a_ value is influenced by further substituents. Therefore, the acidity of the ammonium groups of ω-aminoethylcellulose carbamate [23], (3-ethoxy-3-oxopropyl)(2-aminoethyl)cellulose carbamate **6** and the polyzwitterion **7a** were compared. Polycation **6** was titrated forth and back in range from pH 4.3 to 10 (Figure 3). The rising point of inflection, representing the p*K*_a_ value of the amino group (9.0), was determined by the first derivative of the curve (dpH/dV) that yields the minimum. To obtain the p*K*_a_ value of the amino group in the polyzwitterionic molecule the titration curve of **7a** could be considered. In Figure 4 the minimum of the first derivative at 340 μL indicates a p*K*_a_ value of 9.7. Thus, the p*K*_a_ increases from 8.6 [23] to 9.0 if the ester moiety is present within the polymer chain and, moreover, the value increases to 9.7 for cellulose carbamate containing carboxylate. The higher affinity of the protons to the polymer chain may be explained by hydrogen bonding to the carboxylic functionalities. The p*K*_a_ value of the carboxylic group is 4.0 and could be obtained from the minimum at 230 μL (Figure 4).

To determine the isoelectric point (IP) of the polyzwitterion, the falling point of inflection between the p*K*_a_ values has to be considered. In Figure 4 the maximum of dpH/dV at 285 μL indicates an IP of 7.3. Naturally, the summation of the charges of the functional groups has to yield a neutral molecule at the IP and a ratio of carboxylic groups to amino groups of 0.997 was predicted from IP and p*K*_a_ values. The consumption of sodium hydroxide solution leads to an equal partial DS_β-alanine_ and DS_EDA_ of 0.53. The decrease of the DS values compared to the protected polyzwitterion **4** could be explained by a reduced accessibility of the functional groups in aqueous solution or the cleavage of carbamate moieties from the polymer backbone during acidic and basic treatment. However, the ratio between amino and carboxylic groups is balanced. In addition, the IP was proofed by rheology. The IP is indicated by a minimum of the relative viscosity of a polyzwitterion solution at pH value of 7.7 (Supporting Information File 1).

#### Precipitation and self-complexation

**Figure 5:**
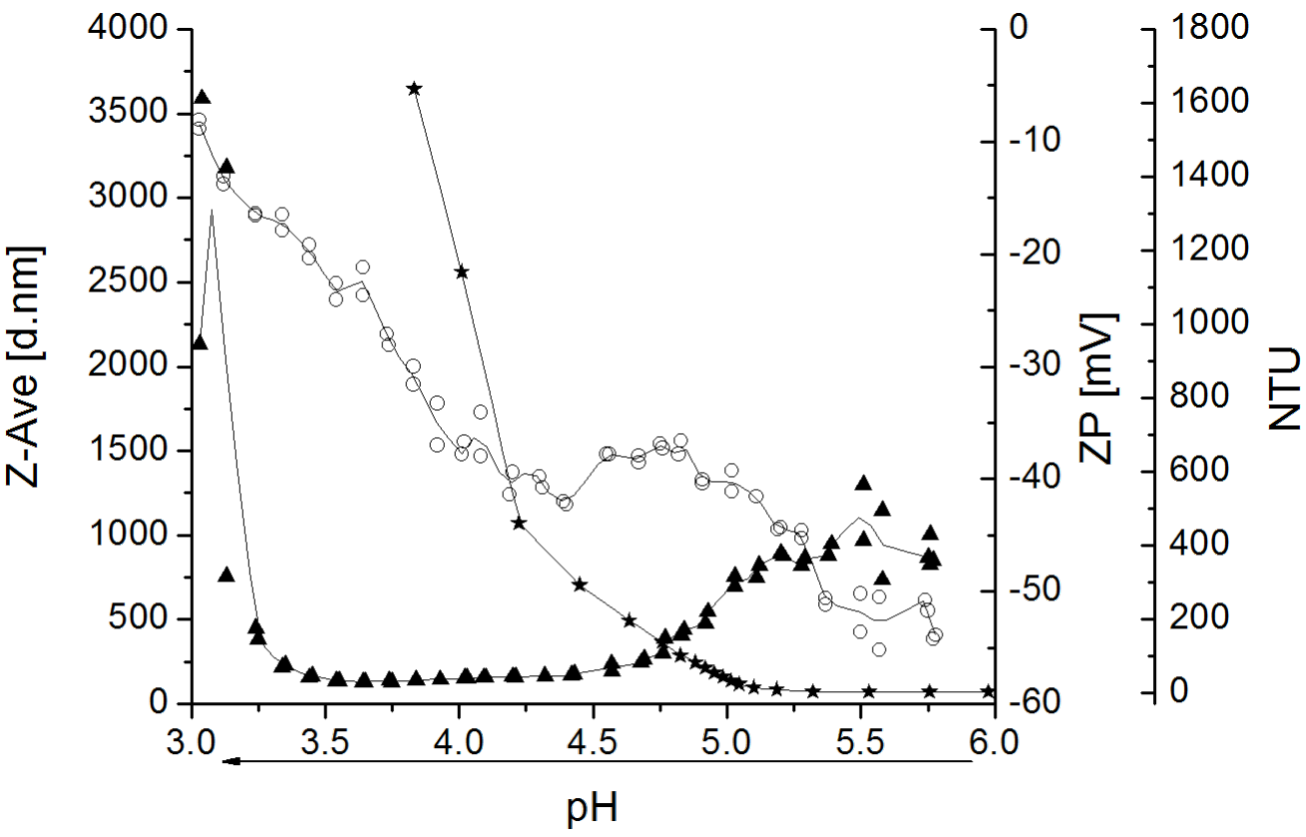
Acid–base titration of (2-carboxyethyl)(*N*-Boc-2-aminoethyl)-cellulose carbamate (**5**, 0.1%) from pH 6 to pH 3 (arrow): turbidity (nephelometric turbidity units, NTU 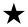), Z-average (d. nm ▲), zeta potential (ZP, mV ○), and interpolation (line) determined by nephelometry and dynamic light scattering (DLS).

The pH value influences the solution state of polyanions, polycations, or polyzwitterions. While the polycation **6** is soluble in water at the whole pH range from 3 to 11, aqueous solutions of polyanion **5** show precipitation below pH values of 5 due to the protonation of the carboxylate and the hydrophobic Boc group. Figure 5 reveals an increase of turbidity with decreasing pH value. Moreover, dynamic light scattering (DLS) display details about the aggregates formed. The measurements of dissolved polymer and aggregates with the DLS equipment yield no useful results, but from pH 4.5 to 3.5 nanoparticles with a Z-average diameter of about 200 nm could be detected. During the titration, the zeta potential of the particles increases from −40 to −10 mV according to the protonation of the carboxylate and thus, the compensation of the negative charge of the boundary surface.

**Figure 6:**
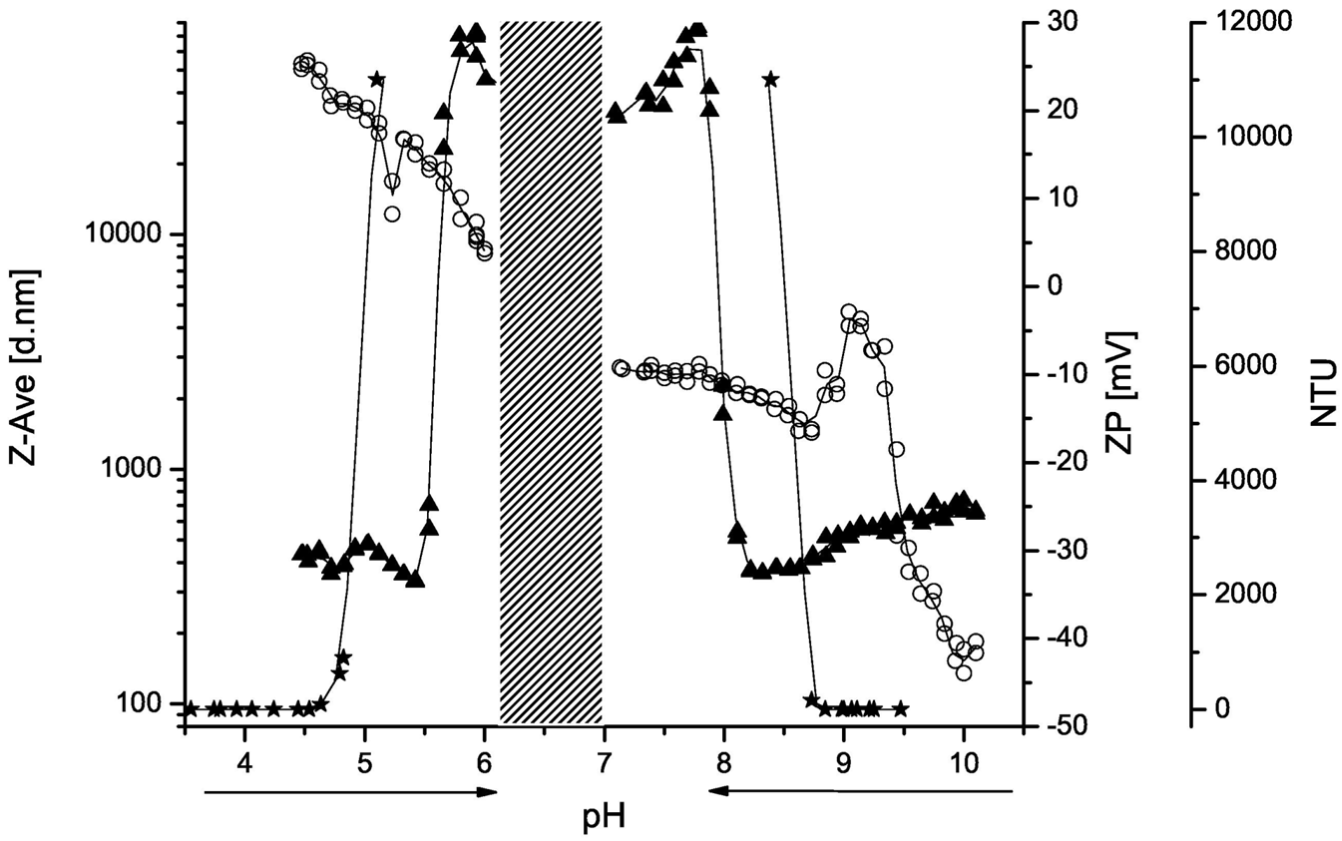
Acid–base titration of (2-carboxyethyl)(2-aminoethyl)cellulose carbamate (**7a**, 0.4% in water) from pH 3.5 to pH 6 and from pH 10 to pH 7 (arrows): turbidity (nephelometric turbidity units, NTU 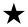), Z-average (d. nm ▲), zeta potential (ZP, mV ○), and interpolation (line) determined by nephelometry and dynamic light scattering (DLS).

Polyzwitterion **7a** shows self-complexation at neutral pH value if no salt is added. Figure 6 illustrates the titration from acidic and alkaline solution and the gap of solubility. In the border from solubility to insolubility at pH value of 5 and 8.5, the turbidity and the Z-average diameter increase significantly. In the pH scale from 4.5 to 6, the zeta potential decreases from +25 to +5 mV due to the deprotonation of the carboxylic moiety. In alkaline solution the zeta potential increases with decreasing pH and the amino groups become protonated. The Z-average diameter seems to decrease with increasing protonation. However, aggregates of widely distributed sizes (high polydispersity) become regular particles due to the electrostatic stabilization indicated by an increase of the absolute value of the zeta potential at pH value of 9. At lower pH values, large aggregates are formed induced by self-complexation.

#### Polyelectrolyte complex formation with polyDADMAC

**Figure 7:**
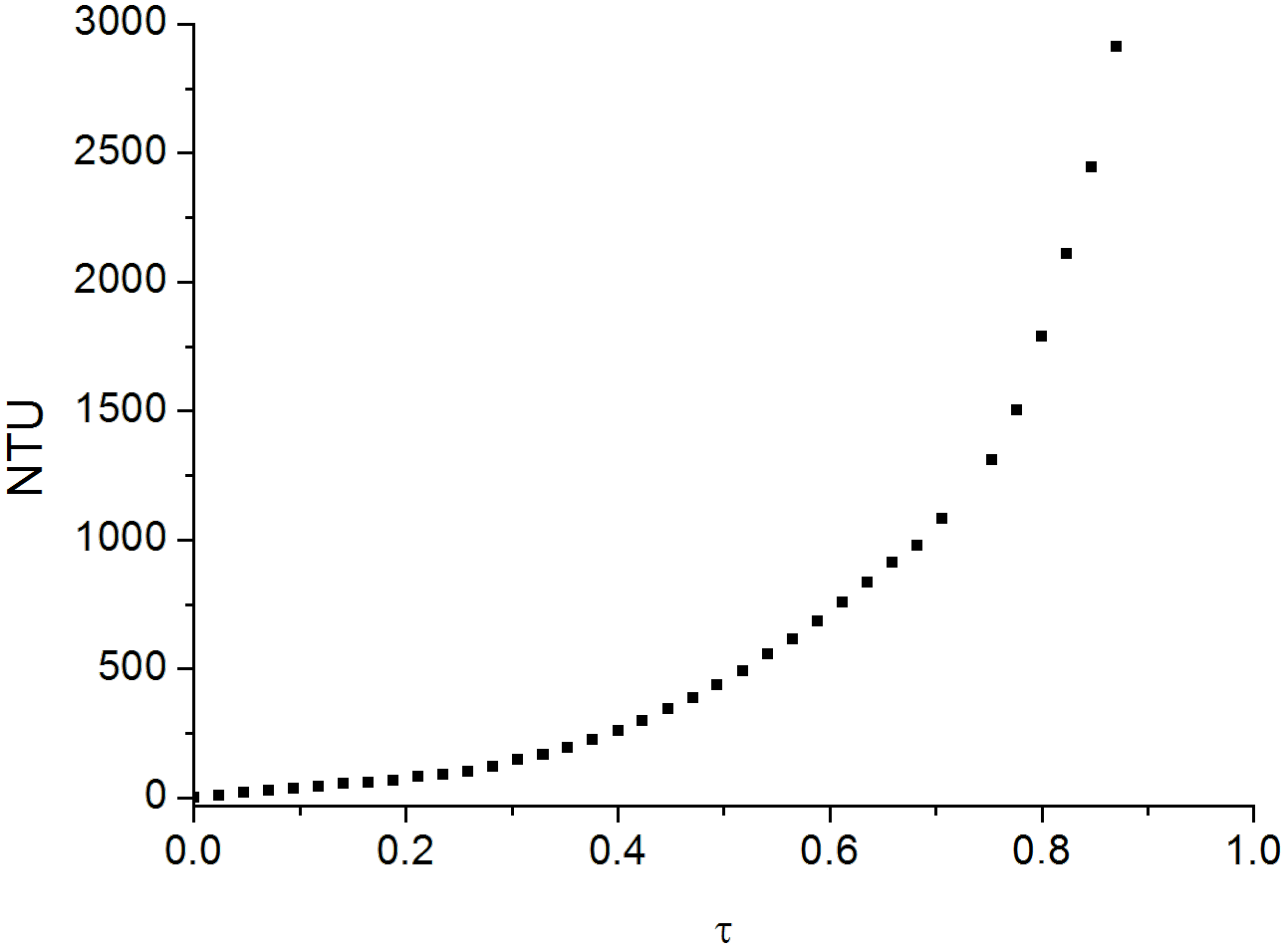
Titration of (2-carboxyethyl)(2-aminoethyl)cellulose carbamate (**7a**, 0.1% in water) at pH 11.5 with polydiallyldimethylammonium chloride (polyDADMAC); turbidity (nephelometric turbidity units, NTU) in dependence on degree of titration (τ).

**Figure 8:**
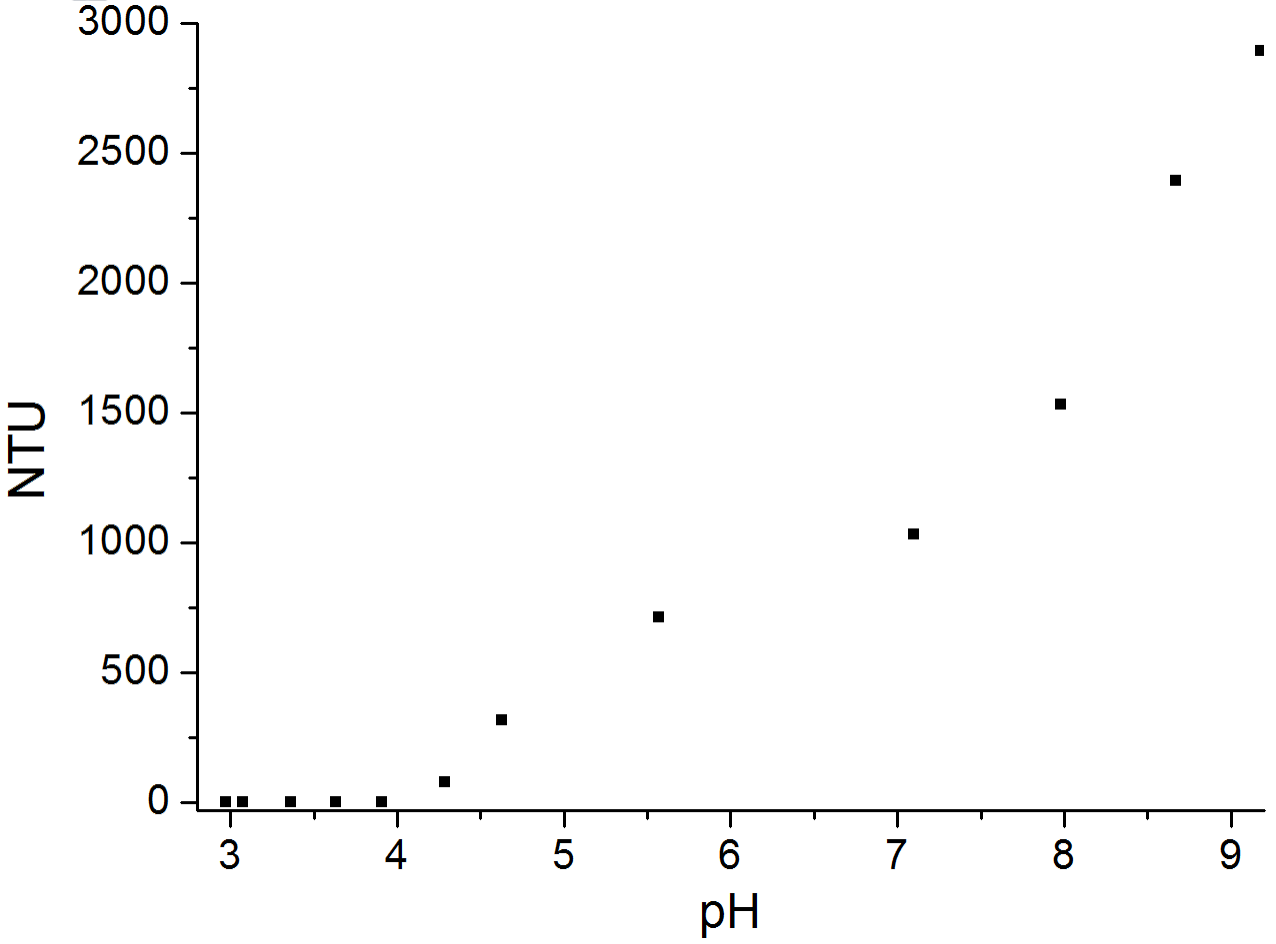
Titration of (2-carboxyethyl)(2-aminoethyl)cellulose carbamate (**7a**, 0.1% in water) and polyDADMAC (equimolar to carboxylic groups) with sodium hydroxide solution; turbidity (nephelometric turbidity units, NTU) in dependence on pH value.

A permanently charged polycation (polydiallyldimethylammonium chloride, polyDADMAC) was applied to study the formation of interpolyelectrolyte complexes. In the first experiment a solution of the zwitterionic cellulose derivative **7a** was adjusted to pH 11.5 in order to yield the maximum of negative charges. The addition of polycation causes the formation of particles resulting in an increased turbidity, finally forming macroscopic aggregates (Figure 7). The degree of titration (τ) is defined by the ratio of the amount of polyDADMAC and the amount of carboxylate in the cellulose derivative. Moreover, a solution of **7a** and polyDADMAC (τ = 1) was titrated with aqueous sodium hydroxide solution (Figure 8). In acidic medium, cationic charges are predominant and repulsive interaction of the polymer chains lead to a clear solution. At pH value from 4 to 5 an increase in turbidity indicates the attractive forces between the molecules due to the deprotonation of the carboxylic groups. Thus, the formation of interpolyelectrolyte complexes is switchable in a physiological relevant range of the pH value.

## Conclusion

In this work a well soluble cellulose-based polyzwitterion with weak ionic groups could be efficiently synthesized starting from cellulose phenyl carbonate. It became possible to access the polyanion, the polycation, and the polyzwitterion by the orthogonal removal of protecting groups. While IR spectroscopy could not proof the presence of all functional groups, the molecular structure was clearly described by NMR spectroscopy. Potentiometric titration (pH), nephelometry, rheology and dynamic light-scattering revealed physicochemical characteristics of the cellulose derivatives. Acid dissociation constants, isoelectric point and complexation behavior are in good agreement and thus, a well characterized zwitterionic cellulose carbamate with excellent properties was discovered. Preliminary studies show the formation of pH-responsive interpolyelectrolyte complexes applying polydiallyldimethylammonium chloride. The particles are switchable in a physiological relevant range of pH values and are promising nanocarriers in the field of drug delivery that will be a subject of further studies.

## Experimental

### Materials

Microcrystalline cellulose (Avicel PH-101) with a DP_w_ of 330 was purchased from Sigma-Aldrich and dried at 60 °C in vaccum. Polydiallyldimethylammonium chloride (Sigma-Aldrich) with a very low molecular weight of *M*_w_ < 100000 g/mol was used as received. Other chemicals and solvents were purchased from Sigma-Aldrich, Acros Organics or TCI Europe and were used without further treatment.

Cellulose phenyl carbonate was prepared by esterification of cellulose, dissolved in *N*,*N*-dimethylacetamide (DMAc)/LiCl, applying pyridine and phenyl chloroformate [21]. **1**, DS 1.92 (determined by means of ^1^H NMR spectroscopy after peracetylation), FTIR (KBr): 1770 cm^−1^ (ν_C=O_); ^13^C NMR (63 MHz, DMSO-*d*_6_) δ 153.4, 153.1, 151.5 (C=O), 151.1 (C-Ph), 130.0 (CH-Ph), 126.6 (CH-Ph), 121.5 (CH-Ph), 103.0 (C-1) 99.2 (C-1'), 82-67 (C-2, C-3, C-4, C-5, C-6) ppm.

*N*-*tert*-Butoxycarbonyl-1,2-ethanediamine (**3**) was obtained according to the procedure described by Krapcho and Kuell [24] and purified by distillation in vaccum (7 mbar, 109 °C). Yield: 67% ^1^H NMR (250 MHz, CDCl_3_) δ 5.17 (bs, NH), 3.07 (m, CH_2_), 2.71 (m, CH_2_), 1.35 (s, CH_3_), 1.12 (s, NH_2_) ppm; ^13^C NMR (63 MHz, CDCl_3_) δ 156.2 (C=O), 79.0 (CMe_3_), 43.4 (CH_2_), 41.8 (CH_2_), 28.3 (CH_3_) ppm.

### Measurements

NMR spectra were acquired on a Bruker Avance 400 MHz (Bruker Biospin, Rheinstetten, Germany) with 16 scans and 25 mg sample per mL solvent for ^1^H NMR spectroscopy (room temperature) and up to 200,000 scans for ^13^C NMR spectroscopy (70 °C) applying up to 100 mg sample per mL solvent. FTIR spectra were recorded on a Nicolet AVATAR 370 DTGS spectrometer (Thermo Scientific, Schwerte, Germany) with the KBr technique. Elemental analyses were performed by a CHNS 932 Analyzer (Leco, Mönchengladbach, Germany). Nephelometric measurements were carried out with a Turbiquant^®^ 3000 IR from Merck (Darmstadt, Germany). The self complexation at different pH values was investigated by potentiometric titration with 0.25 M hydrochloric acid and 0.25 M sodium hydroxide solution using the Zetasizer Nano ZS from Malvern Instruments (Malvern, UK) equipped with a MPT-2 autotitrator. Z-Average diameter and zeta potential were determined by dynamic light scattering (DLS) and obtained by the cumulants method assuming spherical shape. Each measurement was repeated at least two times. Manual potentiometric titrations were performed with a SevenMulti™ pH meter (Mettler Toledo, Gießen, Germany). The viscosity of the polymer solutions was measured with a Haake Mars II cone-plate rheometer (Thermo Scientific, Schwerte, Germany) in controlled rate mode at 20 °C. Shear rates were varied from 0.1 to 1000 s^−1^ in a cycle of increasing and decreasing shear rate over 5 min. The linear range was extrapolated to zero shear viscosity.

### Syntheses

#### Synthesis of (3-ethoxy-3-oxopropyl)(*N*-Boc-2-aminoethyl)cellulose carbamate **4**

In a centrifuge tube β-alanine ethyl ester hydrochloride (11.27 g, 73 mmol) dissolved in 70 mL *N*,*N*-dimethylformamide (DMF) and a solution of triethylamine (5.93 g, 59 mmol) in 20 mL DMF was added. The tube was shaken immediately and allowed to stand for 1 h at room temperature. The precipitate was removed by centrifugation and the clear solution of β-alanine ethyl ester (**2**) was mixed with *N*-*tert*-butoxycarbonyl-1,2-ethanediamine (**3**, 11.73 g, 73 mmol), dissolved in 60 mL DMF.

The solution of the amines **2** and **3** was added rapidly under vigorous stirring to cellulose phenyl carbonate **1** (10 g, DS 1.92, 49 mmol carbonate) dissolved in 150 mL DMF. The reaction mixture was allowed to react for 24 h at 60 °C. After precipitation into 2.5 L of water, the product was isolated by filtration. The product was washed three times with 1 L of water and dried in vacuum at 40 °C. Yield: 90%, DS_β-alanine ester_ 0.88, DS_Boc-EDA_ 0.95 (determined by elemental analysis from nitrogen content assuming a total DS of 1.83 according to a conversion of carbonate of 95% [21]), FTIR (KBr): 1716 cm^−1^ (ν_C=O_, carbamate and ester); ^13^C NMR (100 MHz, MeOD) δ 172.0 (C=O, ester), 157.0 (C=O, carbamate), 103.0 (C-1), 101.4 (C-1'), 83–74 (C-2, C-3, C-4, C-5), 78.8 (CMe_3_), 63.0 (C-6), 60.4 (OCH_2_), 40.5 (CH_2_), 39.9 (CH_2_), 36.5 (CH_2_), 34.1 (CH_2_), 27.5 (C(CH_3_)_3_), 13.2 (CH_3_) ppm.

#### Synthesis of (2-carboxyethyl)(*N*-Boc-2-aminoethyl)cellulose carbamate sodium salt (**5**)

A solution of **4** (1 g) in 15 mL methanol was mixed with 2 g sodium hydroxide dissolved in 5 mL water under nitrogen atmosphere and allowed to react for 1 h at room temperature under stirring. The product was isolated by precipitation into 40 mL 2-propanol and subsequent filtration. The precipitate was washed four times with 2-propanol, pre-dried in vacuum at room temperature, dissolved in 10 mL water, dialyzed against deionized water, and finally lyophilized. Yield: 873 mg (88%), FTIR (KBr): 1705 cm^−1^ (ν_C=O_, carbamate), 1581 cm^−1^ (ν_C=O_, carboxylate); ^13^C NMR (100 MHz, D_2_O) δ 180.2 (C=O, carboxylate), 158.0 (C=O, carbamate), 103.0 (C-1), 101.1 (C-1'), 85–66 (C-2, C-3, C-4, C-5), 80.9 (CMe_3_), 63.0 (C-6), 40.3 (CH_2_), 39.8 (CH_2_), 37.9 (CH_2_), 37.3 (CH_2_), 27.8 (C(CH_3_)_3_) ppm.

#### Synthesis of (3-ethoxy-3-oxopropyl)(2-aminoethyl)cellulose carbamate hydrochloride **6**

**4** (2 g) was dissolved in 60 mL 1,4-dioxane at 60 °C. After cooling to room temperature, a flow of hydrogen chloride was bubbled through the solution for 2 h under stirring. The precipitate was isolated by centrifugation, washed one times with 50 mL dioxane, three times with 50 mL 2-propanol and pre-dried in vacuum at room temperature. The half amount of the product was dissolved in 10 mL water, dialyzed against deionized water and finally lyophilized. Yield: 82%, DS_β-alanine ester_ 0.90, DS_EDA_ 0.80 (determined by elemental analysis from nitrogen- and chlorine content), FTIR (KBr): 1716 cm^-1^ (ν_C=O_, carbamate and ester); ^13^C NMR (100 MHz, D_2_O) δ 174.2 (C=O, ester), 157.5 (C=O, carbamate), 102.7 (C-1), 101.0 (C-1'), 82–66 (C-2, C-3, C-4, C-5), 63.0 (C-6), 61.8 (OCH_2_), 39.5 (CH_2_), 38.1 (CH_2_), 36.5 (CH_2_), 34.4 (CH_2_), 13.5 (CH_3_) ppm.

#### Synthesis of (2-carboxyethyl)(2-aminoethyl)cellulose carbamate hydrochloride

**7a**: **5** (0.5 g) was dissolved in 5 mL TFA under stirring for 15 min. After precipitation into 40 mL cold 2-propanol, the product was immediately isolated by centrifugation, washed four times with 25 mL 2-propanol and pre-dried in vacuum at room temperature. The product dissolved in 5 mL water was dialyzed against saturated sodium chloride solution and deionized water, and finally lyophilized. Yield: 340 mg (82 %), DS_β-alanine_ 0.53, DS_EDA_ 0.53, FTIR (KBr): 1716 cm^-1^ (ν_C=O_, carbamate and carboxylic acid); ^13^C NMR (100 MHz, D_2_O) δ 178.7 (C=O, carboxylic acid), 157.6 (C=O, carbamate), 102.6 (C-1), 100.9 (C-1'), 83–67 (C-2, C-3, C-4, C-5), 63.0 (C-6), 39.4 (CH_2_), 38.0 (CH_2_), 37.3 (CH_2_), 36.1 (CH_2_) ppm.

**7b**: 10 mL sodium hydroxide solution (2 g NaOH/10 mL water) was added to the half amount of the product **6** (see procedure above) dissolved in 10 mL water under nitrogen atmosphere. The reaction solution was allowed to react for 1 h at room temperature under stirring. Subsequently, an equimolar amount of hydrochloric acid (50 mmol, 4.2 mL 37%) diluted with 5 mL water was added under cooling with ice and the mixture was precipitated into 200 mL 2-propanol. The product was isolated by centrifugation, washed three times with 50 mL 2-propanol, dried in vacuum at room temperature, dissolved in 10 mL water, dialyzed against deionized water, and finally lyophilized. Yield: 71%

## Supporting Information

File 1 Relative viscosity of an aqueous solution of cellulose carbamate **7a** in dependence on pH value.
