# Supplementary material for: A promising cellulose-based polyzwitterion with pH-sensitive charges
Source: Beilstein J Org Chem. 2014 Jul 8;10:1549–56. doi: 10.3762/bjoc.10.159 (PMC4142972; doi:10.3762/bjoc.10.159)
Supplement: File 1 — Relative viscosity of an aqueous solution of cellulose carbamate 7a in dependence on pH value. [file Beilstein_J_Org_Chem-10-1549-s001.pdf]

Supporting Information

for

A promising cellulose-based polyzwitterion with pH-sensitive charges

Thomas Elschner and Thomas Heinze*

Address: Center of Excellence for Polysaccharide Research, Institute for Organic Chemistry and Macromolecular Chemistry, Friedrich Schiller University of Jena, Humboldtstraße 10, D-07743 Jena, Germany

Email: Thomas Heinze - thomas.heinze@uni-jena.de

* Corresponding author

Relative viscosity of an aqueous solution of cellulose carbamate 7a in dependence on pH value

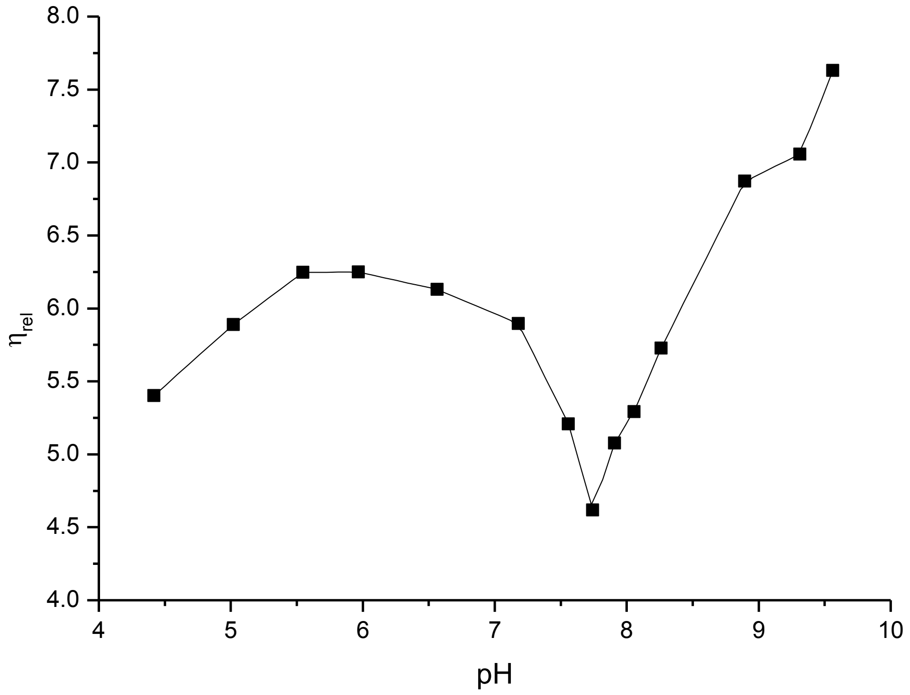

Figure S1: Relative viscosity (η_{rel}) of an aqueous solution of (2-carboxyethyl)(2-aminoethyl)-cellulose carbamate (**7a**, 2% in 0.2 M aqueous NaCl) in dependence on pH value determined by rotational viscometry.
